# Phyto-Functionalized Silver Nanoparticles Derived from Conifer Bark Extracts and Evaluation of Their Antimicrobial and Cytogenotoxic Effects

**DOI:** 10.3390/molecules27010217

**Published:** 2021-12-30

**Authors:** Irina Macovei, Simon Vlad Luca, Krystyna Skalicka-Woźniak, Liviu Sacarescu, Petronela Pascariu, Alina Ghilan, Florica Doroftei, Elena-Laura Ursu, Cristina Mihaela Rimbu, Cristina Elena Horhogea, Cristina Lungu, Gabriela Vochita, Alina Diana Panainte, Constantin Nechita, Maria Andreia Corciova, Anca Miron

**Affiliations:** 1 Faculty of Pharmacy, Grigore T. Popa University of Medicine and Pharmacy, 700115 Iasi, Romania; irina-macovei@umfiasi.ro (I.M.); lungu.cristina@umfiasi.ro (C.L.); alina.gudruman@umfiasi.ro (A.D.P.); maria.corciova@umfiasi.ro (M.A.C.); 2 Biothermodynamics, TUM School of Life Sciences, Technical University of Munich, D-85354 Freising, Germany; vlad.luca@tum.de; 3 Department of Natural Products Chemistry, Medical University of Lublin, 20-093 Lublin, Poland; kskalicka@pharmacognosy.org; 4 Petru Poni Institute of Macromolecular Chemistry, 700487 Iasi, Romania; livius@icmpp.ro (L.S.); dorneanu.petronela@icmpp.ro (P.P.); diaconu.alina@icmpp.ro (A.G.); florica.doroftei@icmpp.ro (F.D.); ursu.laura@icmpp.ro (E.-L.U.); 5 Department of Public Health, Ion Ionescu de la Brad University of Agricultural Sciences and Veterinary Medicine, 700489 Iasi, Romania; chorhogea@uaiasi.ro; 6 NIRDBS, Institute of Biological Research, 700107 Iasi, Romania; gabriela.vochita@icbiasi.ro; 7 Marin Dracea National Institute for Research and Development in Forestry, 725100 Campulung Moldovenesc, Romania; ncincds@gmail.com

**Keywords:** silver nanoparticles, green synthesis, *Picea abies* bark, *Pinus nigra* bark, polyphenols, antimicrobial activity, antimitotic activity, genotoxicity

## Abstract

Silver nanoparticles synthesized using plant extracts as reducing and capping agents showed various biological activities. In the present study, colloidal silver nanoparticle solutions were produced from the aqueous extracts of *Picea abies* and *Pinus nigra* bark. The phenolic profile of bark extracts was analyzed by liquid chromatography coupled to mass spectrometry. The synthesis of silver nanoparticles was monitored using UV-Vis spectroscopy by measuring the Surface Plasmon Resonance band. Silver nanoparticles were characterized by attenuated total reflection Fourier transform infrared spectroscopy, Raman spectroscopy, dynamic light scattering, scanning electron microscopy, energy dispersive X-ray and transmission electron microscopy analyses. The antimicrobial and cytogenotoxic effects of silver nanoparticles were evaluated by disk diffusion and *Allium cepa* assays, respectively. *Picea abies* and *Pinus nigra* bark extract derived silver nanoparticles were spherical (mean hydrodynamic diameters of 78.48 and 77.66 nm, respectively) and well dispersed, having a narrow particle size distribution (polydispersity index values of 0.334 and 0.224, respectively) and good stability (zeta potential values of −10.8 and −14.6 mV, respectively). Silver nanoparticles showed stronger antibacterial, antifungal, and antimitotic effects than the bark extracts used for their synthesis. Silver nanoparticles obtained in the present study are promising candidates for the development of novel formulations with various therapeutic applications.

## 1. Introduction

Silver nanoparticles (AgNPs) are characterized by being at least one dimension smaller than 100 nm and having a high surface area to volume ratio. Due to their remarkable antimicrobial effects, good optical properties, low thermal and electrical resistance, AgNPs have applications in various sectors such as healthcare, food industry, environmental health, electronics, instrumental analysis, and catalysis [1]. AgNPs can be produced by physical (evaporation and condensation, laser ablation, spray pyrolysis), chemical (chemical reduction, electromagnetic radiation, and electrochemical cell methods), biological (plant extract and microbial reduction) and mechanical (ball milling) methods [1,2]. Both physical and chemical methods have several drawbacks, such as high energy consumption, expensive equipment, long reaction time, use of toxic chemicals and generation of chemical wastes (unreacted chemicals) which have to be discarded [1]. In recent years, biological methods (green synthesis), using either plant extracts or living microorganisms, have gained increased attention as they are simple, rapid, and cost-effective [1,2]. The use of plant extracts is more advantageous for several reasons. First, plants are easily available and inexpensive. Second, in contrast to the aforementioned methods, plant extract mediated synthesis of AgNPs generates waste which is not harmful for the environment. In addition, plant extracts contain various phytochemicals which can act as reducing and/or capping agents in AgNPs synthesis [3]. Phytochemicals in plant extracts are able to reduce Ag ions to Ag atoms and, moreover, to form an outer shell around nanosized metallic Ag, thus averting the aggregation of nanoparticles and stabilizing them [4]. Plant extracts act not only as reducing and/or capping agents, but also as modulators of the biological effects of nanosized metallic Ag [5]. Thus, AgNPs synthesized using plant extracts or pure phytochemicals have been reported to develop a wide range of biological activities, such as antibacterial, antifungal and antiviral [6], anti-inflammatory [7], antioxidant [5,7], wound healing [8], anticancer [1,3], anticoagulant [9], cardioprotective [10], liver protective [10], antidiabetic [11], anti-cataractogenic [12] and anti-ageing [13] activities.

Conifer bark is one of the best candidates for AgNPs synthesis. It contains various biologically active compounds such as polyphenols (phenolic acids, flavonoids, proanthocyanidins, stilbenes, lignans), terpenoids, sterols, carbohydrates which act as reducing and/or stabilizing agents in AgNPs synthesis [14]. Proanthocyanidins (condensed tannins), major components in conifer bark, are oligomers and polymers of flavan-3-ol units. There are several types of proanthocyanidins depending on the monomer. Procyanidins, prodelphinidins and propelargonidins are the most common, the monomer being (epi)catechin, (epi)gallocatechin and (epi)afzelechin, respectively [15]. Proanthocyanidins from conifer bark have been reported to develop antioxidant, anti-inflammatory, immunomodulatory, anticancer, and hypoglycemic effects. They also have positive effects on the cardiovascular (vasorelaxation, activation of microcirculation, strengthening of capillaries, reduction of blood pressure and atherosclerotic lesions) and nervous (neuroprotection) systems [14,16]. The main stilbenes identified in conifer bark are pinosylvin, piceatannol, *trans*-resveratrol, isorhapontigenin and pinostilbene along with their glycosides (astringin, resveratroloside, piceid, isorhapontin, pinostilbenoside) [14]. Known as phytoalexins (usually produced by plants in response to pathogen attack, UV, or ozone exposure, cold, drought), stilbenes display a broad spectrum of biological activities (antimicrobial, antioxidant, anti-inflammatory, chemopreventive, lipolytic). Numerous studies support their role in the protection against cardiovascular and neurodegenerative disorders and cancer [14,17,18]. Conifer bark contains a wide range of flavonoids, quercetin, kaempferol, naringenin, eriodictyol and their derivatives being the most common ones [14]. Flavonoids are undoubtedly the class of plant metabolites having the most numerous health benefits. Among flavonoids found in conifer bark, taxifolin (dihydroquercetin) has gained great interest due to its excellent anti-inflammatory, antioxidant and vasoprotective activities which provide beneficial effects in various diseases (cardiovascular, liver, skin and cognitive disorders, malignancies) [19]. Overall, the use of conifer bark extracts in AgNPs synthesis might result in phyto-functionalized AgNPs with promising therapeutic applications.

Although conifer bark is a rich source of compounds having reducing abilities and exerting a broad range of bioactivities, its potential application in the green synthesis of AgNPs has been scarcely investigated [20,21]. In this context, the present study was initiated; the study reports on the green synthesis of colloidal AgNPs solutions using aqueous extracts derived from the bark of two conifer species, *Picea abies* (L.) H. Karst. (Norway spruce) and *Pinus nigra* J. F. Arnold (black pine). The aqueous colloidal AgNPs solutions were further subjected to physicochemical characterization by various spectroscopic and microscopic methods. Their antimicrobial, antimitotic and genotoxic effects were also evaluated. 

## 2. Results and Discussion

### 2.1. Phenolic Content and Profile of Conifer Bark Extracts

**Figure 1:**
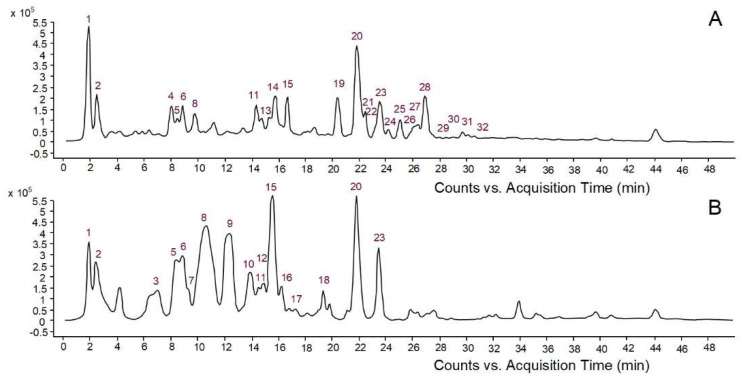
Base peak chromatograms of *Picea abies* (**A**) and *Pinus nigra* (**B**) aqueous bark extracts.

In this study, aqueous bark extracts of two conifers, namely *Picea abies* and *Pinus nigra*, were used as reducing and stabilizing agents to synthesize AgNPs. Conifer bark is a rich source of phenolic compounds with very diverse chemical structures [14]. According to previous reports, phenolic compounds play a major role in the formation and stabilization of AgNPs, acting as reducing and stabilizing agents [4,5,22,23]. Compounds having at least two phenolic hydroxyl groups (*ortho* or *para* position) efficiently reduce Ag ions to Ag atoms, the phenolic hydroxyls being oxidized into quinones; the quinone-type molecules cover the surface of AgNPs, thus enhancing their stability [4,23]. Other functional groups in polyphenols, such as the carboxyl moieties of phenolic acids, might also interact with Ag surface contributing to the stabilization of AgNPs [23]. Therefore, the first objective of the present study was to investigate the quantitative and qualitative phenolic composition of aqueous extracts derived from *Picea abies* and *Pinus nigra* bark. *Picea abies* bark extract showed higher contents of total phenolics (10.19 ± 0.15 vs. 4.51 ± 0.34 mg gallic acid/mL extract) and proanthocyanidins (5.06 ± 0.49 vs. 0.95 ± 0.05 mg cyanidin/mL extract) than *Pinus nigra* bark extract. The phenolic profiles of bark extracts are illustrated in Figure 1. High-performance liquid chromatography with diode array detection coupled to electrospray ionization quadrupole time-of-flight tandem mass spectrometry (HPLC-DAD-ESI-Q-TOF-MS/MS) revealed 24 compounds in *Picea abies* bark extract and 17 compounds in *Pinus nigra* bark extract. Simple phenolics, benzoic acid derivatives, hydroxycinnamic acid derivatives and other phenylpropanoids, flavonoids, proanthocyanidins (procyanidin oligomers) and stilbenes were tentatively identified by comparing their spectral data with those reported in databases (METLIN, KNApSacK, PubChem, NIST Chemistry WebBook) or literature (Table 1).

### 2.2. Synthesis of AgNPs

**Figure 2:**
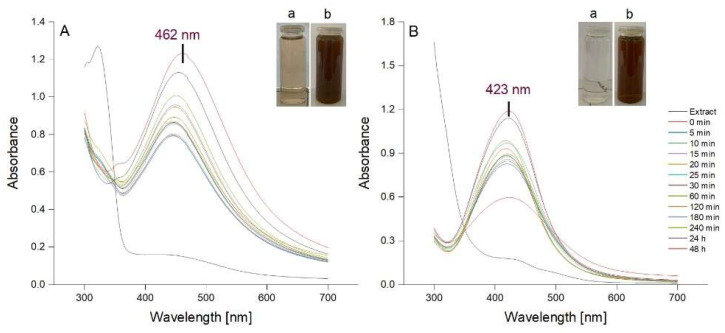
UV-Vis absorption spectra of AgNPs colloidal solutions synthesized using *Picea abies* (**A**) and *Pinus nigra* (**B**) aqueous bark extracts (a: extract + 1 mM AgNO_3_ at time 0; b: extract + 1 mM AgNO_3_ at 60 min).

AgNPs were synthesized using a previously described method consisting, in brief, in stirring each aqueous bark extract with AgNO_3_ (1 mM) for 60 min at room temperature [40]. The synthesis of AgNPs was assessed spectrophotometrically, the spectrum of each reaction mixture being recorded at different intervals during 60 min stirring and post-stirring (1, 2, 3, 24 and 48 h). The formation of AgNPs is indicated by a visible colour change of the reaction mixture caused by the reduction of Ag^+^ to Ag^0^ and concomitant oxidation of the phenolic moieties into quinones. In addition, a Surface Plasmon Resonance (SPR) band occurs between 420 and 500 nm due to the collective oscillations of metal free electrons [4,40,41]. In our study, the colour of both reaction mixtures turned from light brown to dark reddish brown after a 60 min reaction time. The UV-Vis spectra of the colloidal solutions of AgNPs, recorded during stirring and post-stirring, are shown in Figure 2. The SPR bands at 462 and 423 nm confirmed the formation of AgNPs by *Picea abies* and *Pinus nigra* bark extracts, respectively. According to the literature, the lower λ_max_ value for *Pinus nigra* bark extract derived AgNPs (423 nm) indicates smaller size nanoparticles [42]. For both extracts, the intensity of SPR bands increased with time tending to stabilize after 24 h, more apparently for *Pinus nigra* bark extract derived AgNPs. The latter sharpened over time with no change in position whereas *Picea abies* bark extract derived AgNPs showed broader SPR bands which slightly shifted from shorter to longer wavelengths over time. Shifting from shorter to longer wavelengths indicates an increase in AgNPs dimensions [42]. At each time interval, both *Picea abies* and *Pinus nigra* bark extract derived AgNPs showed single SPR bands. According to previous studies, single sharp SPR bands suggest that AgNPs have a spherical shape and do not aggregate in large clusters [4,43,44]. Overall, according to the UV-Vis spectroscopic data, 60 min stirring followed by 24 h at rest was established to be the optimal reaction time with respect to the accumulation of AgNPs but also their size and stability. In *Picea abies* and *Pinus nigra* bark extract derived AgNPs solutions, the concentration of AgNPs was calculated to be 6.67 × 10^−10^ and 6.89 × 10^−10^ mol/L, respectively.

### 2.3. Attenuated Total Reflection Fourier Transform Infrared Spectroscopy 

**Figure 3:**
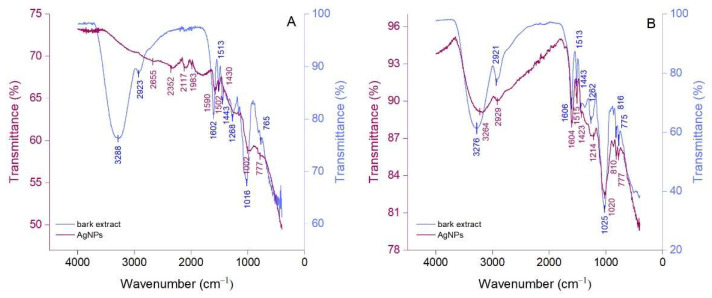
ATR-FTIR spectra of *Picea abies* (**A**) and *Pinus nigra* (**B**) bark extracts and their derived AgNPs.

Attenuated total reflection Fourier-transform infrared (ATR-FTIR) spectroscopy was performed to identify the compounds in bark extracts which are responsible for the reduction of Ag^+^ to Ag^0^ and stabilization of AgNPs. The ATR-FTIR spectra of bark extracts and their derived AgNPs are illustrated in Figure 3. *Picea abies* and *Pinus nigra* bark extracts (Figure 3A,B) showed strong absorption bands at 3288 cm^−1^ and 3276 cm^−1^, respectively, corresponding to the stretching vibrations of O-H groups [45,46]. These bands can be correlated with the presence of polyphenols, carbohydrates, and proteins in bark extracts. The bands at 2923 cm^−1^ and 2921 cm^−1^ (*Picea abies* and *Pinus nigra* bark extracts, respectively) denote the C-H stretching vibration [47]. Bands belonging to the stretching vibrations of the aromatic rings and =C–O–C groups of flavonoids occur around 1600 cm^−1^, 1500 cm^−1^, 1450 cm^−1^ and 1270 cm^−1^ in the spectra of both bark extracts [45]. The band at 1513 cm^−1^ (both extracts) could also be ascribed to the amide II region in proteins [48] whereas the bands at 1016 cm^−1^ (*Picea abies* bark extract) and 1025 cm^−1^ (*Pinus nigra* bark extract) might belong to the C–O–C stretching vibration in carbohydrates [49,50]. The bands at 765 cm^−1^ (*Picea abies* bark extract), 816 cm^−1^ and 775 cm^−1^ (*Pinus nigra* bark extract) denote the aromatic C–H out of plane bending vibrations, the ones at 765 cm^−1^ and 775 cm^−1^ being characteristic for the B ring of flavonoids [51]. For both bark extracts, polyphenols were undoubtedly the major contributors in the green synthesis and stabilization of AgNPs. Apart from polyphenols, carbohydrates and proteins might also have played a role, acting as reducing and/or stabilizing agents [52]. The ATR-FTIR spectra of AgNPs (Figure 3A,B) illustrate the functional groups belonging to the capping/stabilizing molecules which are present on their surface. The typical IR bands in bark extracts are also observed in AgNPs spectra, but they are attenuated and/or shifted suggesting a synergy between polyphenols and other phytochemicals in bark extracts in the stabilization of AgNPs [53,54]. The intensity of the band at 3288 cm^−1^ almost disappeared in the ATR-FTIR spectra of *Picea abies* bark extract-derived AgNPs indicating that O-H groups were almost entirely involved in the reduction of Ag^+^ to Ag^0^.

### 2.4. Raman Spectroscopy Analysis

**Figure 4:**
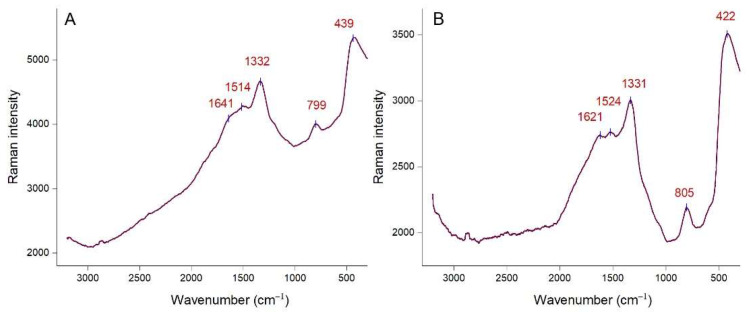
Raman spectra of *Picea abies* (**A**) and *Pinus nigra* (**B**) bark extract derived AgNPs.

The functional groups on the surface of AgNPs were also investigated by Raman spectroscopy. Raman spectra of *Picea abies* and *Pinus nigra* bark extract derived AgNPs (colloidal solutions) are depicted in Figure 4A,B, respectively. Both spectra show intense Raman bands at similar wavenumbers. The bands at 1332 cm^−1^ and 1514 cm^−1^ (Figure 4A), 1331 cm^−1^ and 1524 cm^−1^ (Figure 4B) correspond to the symmetric and asymmetric C=O stretching vibrations [53,55]. Bands at 799 cm^−1^ (Figure 4A) and 805 cm^−1^ (Figure 4B) could be ascribed to the stretching vibration of the glycosidic C–O–C linkage [55]. Amides are also presumed to be present due to the N–H bending vibrations at 439 cm^−1^ (Figure 4A) and 422 cm^−1^ (Figure 4B) [53,56]. Other bands (1641 cm^−1^ in Figure 4A and 1621 cm^−1^ in Figure 4B) are related to the C–C stretching vibrations [55,56]. It is known that polyphenols bind to AgNPs surface through the C=O moieties of quinones (formed by oxidation of phenolic hydroxyl groups) and/or carboxylate groups (in case of phenolic acids) [4,27]. Proteins and acidic carbohydrates also attach to AgNPs through carboxylate groups; in case of proteins, amino groups might also be involved [47,57]. According to Raman spectroscopy data, polyphenols and other phytochemicals (carbohydrates, proteins) are responsible for the stabilization of AgNPs.

### 2.5. Dynamic Light Scattering Analysis

**Figure 5:**
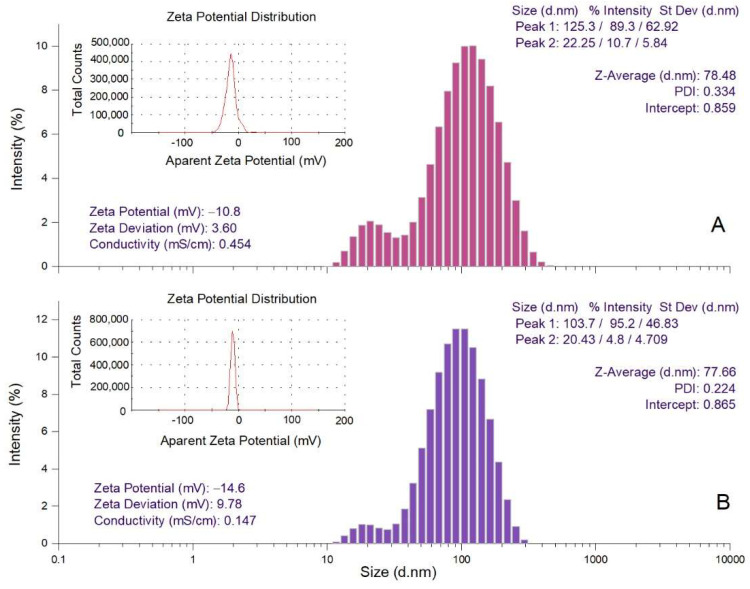
DLS analysis of *Picea abies* (**A**) and *Pinus nigra* (**B**) bark extract derived AgNPs (Z-average—hydrodynamic diameter; PDI—polydispersity index; d.nm—diameter in nm).

The hydrodynamic diameter (Z-average), polydispersity index (PDI) and zeta potential values of *Picea abies* and *Pinus nigra* bark extract derived AgNPs were determined by dynamic light scattering (DLS) analysis (Figure 5). Z-average is the mean hydrodynamic diameter; PDI and zeta potential estimate the width of distribution and stability of AgNPs, respectively [58]. The hydrodynamic diameters of *Picea abies* and *Pinus nigra* bark extract derived AgNPs varied from 22.25 to 125.3 nm (Z-average 78.48 nm) and 20.43 to 103.7 nm (Z-average 77.66 nm), respectively. The values of PDI (0.334 and 0.224 for *Picea abies* and *Pinus nigra* bark extract derived AgNPs, respectively) indicate a narrow particle size distribution. As already mentioned, PDI estimates the homogeneity of size distribution in colloidal AgNPs solutions. PDI values higher than 0.7 denote a broad particle size distribution, lower values indicating better homogeneity [59]. The negative values of zeta potential (−10.8 and −14.6 mV for *Picea abies* and *Pinus nigra* bark extract derived AgNPs, respectively) indicate the stability of the synthesized nanoparticles [43,48]. It is well-known that the electrostatic repulsion between the negatively charged nanoparticles prevents their agglomeration, conferring high dispersity and good stability in time [48,60].

### 2.6. Scanning Electron Microscopy and Energy Dispersive X-ray Analysis

**Figure 6:**
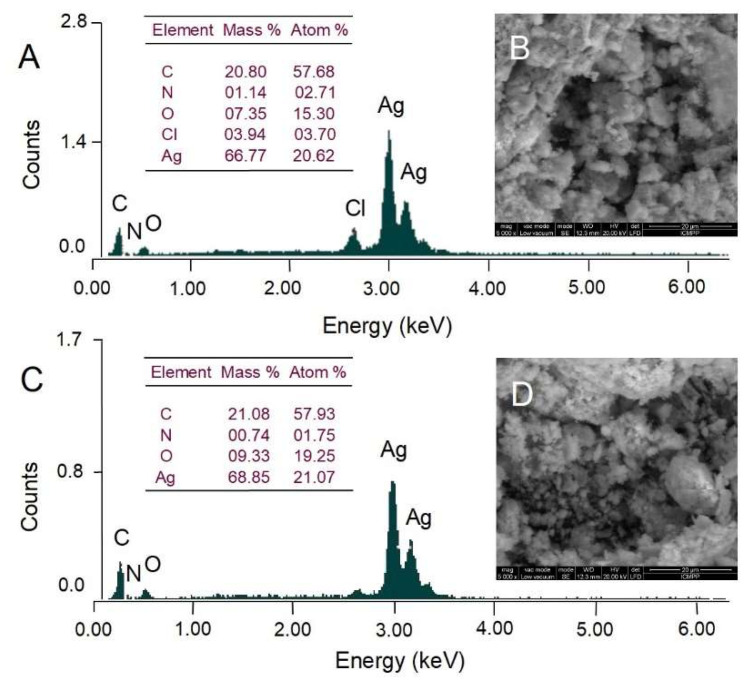
Elemental composition (EDX analysis) and scanning electron microscopy (SEM) micrographs of *Picea abies* (**A**,**B**) and *Pinus nigra* (**C**,**D**) bark extract derived AgNPs.

**Figure 7:**
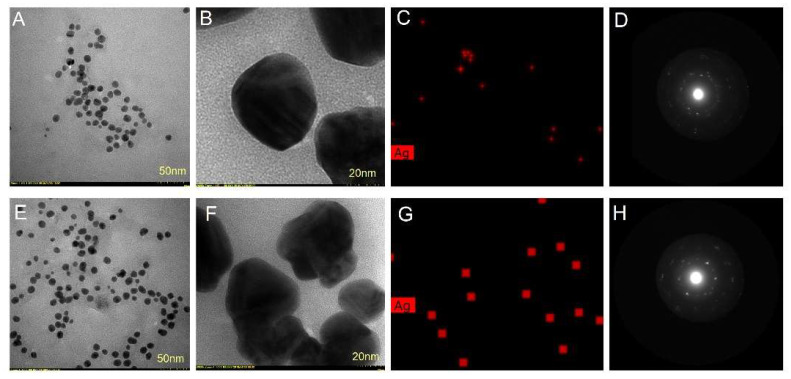
*Picea abies* bark extract derived AgNPs: TEM micrographs (**A**,**B**), TEM-EDX mapping (**C**) and SAED pattern (**D**); *Pinus nigra* bark extract derived AgNPs: TEM micrographs (**E**,**F**), TEM-EDX mapping (**G**) and SAED pattern (**H**).

Scanning electron microscopy (SEM) was used to observe the surface morphology of AgNPs whereas energy dispersive X-ray analysis (EDX) was conducted to identify and quantify the chemical elements found in AgNPs. The surface morphology of AgNPs visualized by SEM is illustrated in Figure 6. Both *Picea abies* and *Pinus nigra* bark extract-derived AgNPs showed irregular agglomerations (Figure 6B,D). As the transmission electron microscopy (TEM) analysis revealed that synthesized AgNPs were spherical in shape and well dispersed (Figure 7), it might be assumed that nanoparticle aggregation occurred during the low vacuum drying in SEM analysis. Both EDX spectra (Figure 6A,C) showed the presence of strong signal of Ag around 3 keV. Weaker signals for other elements (C, O, N, Cl) are also visible. According to the literature data, they belong to the compounds in plant extracts which bind to the surface of AgNPs and stabilize them acting as capping agents [44].

### 2.7. Transmission Electron Microscopy Analysis

The size, shape and morphology of AgNPs were also investigated by TEM analysis. TEM images showed that AgNPs synthesized with both bark extracts had a roughly spherical shape (Figure 7A,B,E,F). Their sizes ranged from 25 to 75 nm and 15 to 55 nm for *Picea abies* and *Pinus nigra* bark extract derived AgNPs, respectively. The nanoparticle diameters measured by TEM were lower than the ones found by DLS. The differences are mainly due to the fact that DLS determines the hydrodynamic diameter, namely the diameter of the particle together with the molecules or ions attached to its surface in solution (hydrated state) whereas TEM measures the size of vacuum–dehydrated particles [47,58,61]. EDX mapping showed the elemental Ag distribution (the red dots) confirming the reduction of Ag^+^ by *Picea abies* (Figure 7C) and *Pinus nigra* (Figure 7G) bark extracts. The selected area electron diffraction (SAED) patterns of *Picea abies* (Figure 7D) and *Pinus nigra* (Figure 7H) bark extract derived AgNPs showed strong crystalline reflections indicating the crystalline nature of AgNPs. In addition, the lattice fringes observed in the high-resolution TEM mode (Figure 7D,H) represent a direct proof of AgNPs crystallinity.

To the best of our knowledge, the use of conifer bark extracts in the green synthesis of AgNPs has hitherto been poorly investigated. The aqueous extract of *Pinus eldarica* bark generated AgNPs of spherical shape and sizes ranging from 10 to 40 nm [62]. A recent study has reported on the synthesis of AgNPs derived from *Picea abies* bark using different experimental protocols than the ones employed in our study. Thereby, the aqueous bark extract was prepared by ultrasound-assisted extraction (70 °C, 30 min); AgNPs were obtained by ultrasound-assisted synthesis at 60 °C for 3 h using different salts (AgNO_3_ or silver acetate, 10 mL of aqueous bark extract mixed with 90 mL of 1 mM AgNO_3_/silver acetate) and different pH values (4 or 9). The resulted AgNPs were spherical and polygonal with average sizes ranging from 44 to 165 nm; AgNPs having a spherical shape and less than 100 nm average size were obtained at pH = 9 [54]. Another method (constant stirring of *Picea abies* aqueous bark extract and 1 mM AgNO_3_, 1:10, 70 °C, pH = 9, 3 h) generated AgNPs with an average diameter of 226 nm [63]. According to the literature data, AgNPs with size below 100 nm are highly active against Gram-positive and Gram-negative bacteria, viruses and other pathogenic microorganisms [64]. In the present study, the protocol used for AgNPs synthesis was simple and provided spherical AgNPs with average sizes below 100 nm, as revealed by DLS and TEM analyses. 

### 2.8. Antimicrobial Activity

The antimicrobial effects of AgNPs and bark extracts used for their synthesis were assessed by the disk diffusion method against Gram-positive (methicillin-susceptible *Staphylococcus aureus* (MSSA), methicillin-resistant *Staphylococcus aureus* (MRSA), *Staphylococcus epidermidis*, *Streptococcus pyogenes*) and Gram-negative (*Escherichia coli*, *Pseudomonas aeruginosa*) bacteria and fungi (*Candida albicans*). The results are depicted in Table 2.

*Picea abies* and *Pinus nigra* bark extract derived AgNPs showed growth inhibition zones comparable with or larger than those developed by gentamicin (10 µg) against *S. aureus* ATCC 33591 and *S. aureus* ATCC 43300, respectively, both MRSA strains. AgNPs also inhibited the growth of *S. pyogenes*, *E. coli* and *P. aeruginosa* whereas bark extracts showed no activity against these bacterial strains. In addition, AgNPs developed larger inhibition zones against *C. albicans* in comparison with bark extracts. It is noteworthy to mention the efficacy of AgNPs against Gram-negative bacteria (*E. coli*, *P. aeruginosa*). The latter have a lipopolysaccharide outer layer (lipids covalently bound to polysaccharides) which considerably reduces the penetration of hydrophobic antibiotics inside bacteria; Gram-negative bacteria thus have less susceptibility to antibiotics than Gram-positive ones [65,66]. Overall, according to the inhibition zone diameters, *Picea abies* and *Pinus nigra* bark extract derived AgNPs exhibited stronger antimicrobial activity against all tested microbial strains than the corresponding bark extracts; both AgNPs and bark extracts were tested at 25 µL. The mechanisms involved in the antimicrobial activity of AgNPs have already been described. In brief, AgNPs bind to the bacterial/fungal cell surface through electrostatic forces and disrupt its integrity penetrating inside the cell. Then, AgNPs slowly release Ag^+^ which triggers the formation of reactive oxygen species. Oxidative stress negatively affects DNA replication and mitochondrial pathways with a reduction in ATP levels and induction of mitochondria-dependent apoptosis. Moreover, Ag^+^ has strong affinity towards the thiol groups of proteins and enzymes, the interaction resulting in the disruption of metabolic reactions and cell death [6,40,66]. Various phytochemicals present on the surface of AgNPs (capping/stabilizing agents) might improve the adhesion of AgNPs to the microbial cells and their antimicrobial efficacy. For example, polyphenols are well-known antimicrobial agents, mainly due to their interaction with various proteins (enzymes, adhesins, cell envelope transport proteins), thus altering the microbial membrane and metabolic pathways [67].

Some of the antimicrobial activity investigated in this study is similar to that reported in previous studies conducted by Tanase et al., where AgNPs prepared from *Picea abies* bark showed good activity against pathogenic bacteria (MSSA, MRSA, *E. coli*, *Klebsiella pneumoniae*, *P. aeruginosa*) [54,63]. However, a comparison with our results is not feasible due to different assays used to evaluate the antibacterial activity (disk diffusion assay in our study, microdilution method in the previous studies).

### 2.9. Cytogenotoxic Activity

**Figure 8:**
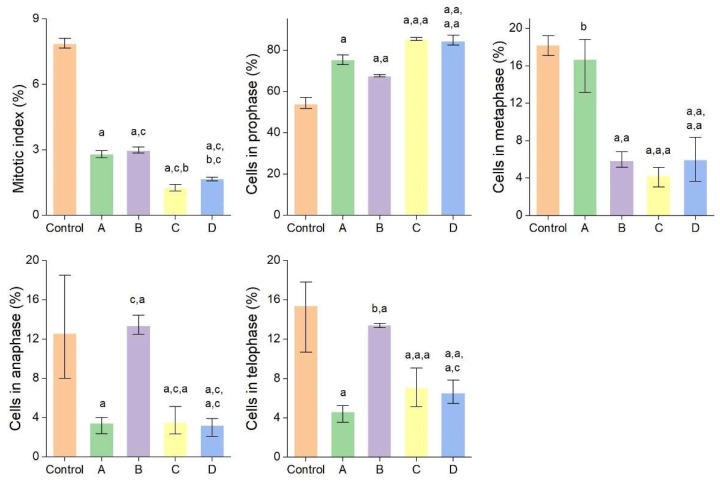
Mitotic index values and percentages of cells in mitosis stages in *Allium cepa* root meristems exposed to *Picea abies* bark extract (**A**), *Pinus nigra* bark extract (**B**), *Picea abies* bark extract derived AgNPs (**C**) and *Pinus nigra* bark extract derived AgNPs (**D**); ^a^: *p* < 0.001; ^b^: *p* < 0.05; ^c^: no significance.

**Figure 9:**
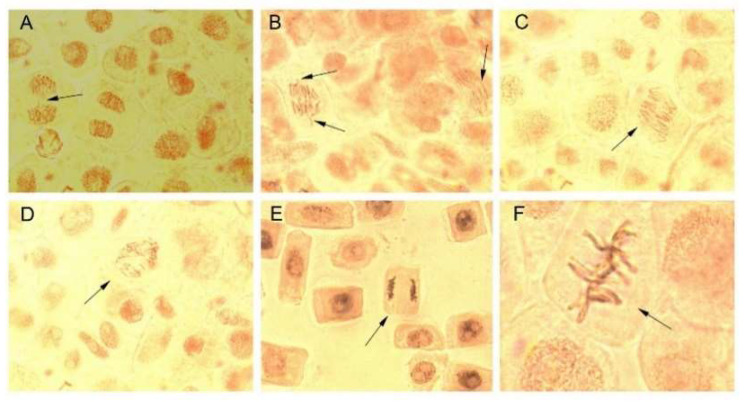
Chromosomal aberrations (**A–C**) and normal stages of mitotic division (**D–F**) in *Allium cepa* root meristems exposed to *Picea abies* and *Pinus nigra* bark extracts and their derived AgNPs. (**A**) telophase with two interrupted bridges (exposure to *Picea abies* bark extract); (**B**) incipient anaphase-telophases with vagrant chromosomes and multiple bridges (exposure to *Picea abies* bark extract derived AgNPs); (**C**) incipient anaphase-telophases with multiple bridges (exposure to *Pinus nigra* bark extract derived AgNPs); (**D**) normal pro-metaphase (control); (**E**) normal anaphase (control); (**F**) normal metaphase (control).

Due to their small size, AgNPs easily penetrate cellular and subcellular structures releasing Ag^+^ responsible for deleterious effects such as increase in oxidative stress, DNA and RNA denaturation, blockage of electron transport chain and ATP formation. AgNPs showed toxicity against different cells and organisms, their toxicity being strongly dependent on size (particles less than 20 nm are highly toxic) and also on the shape, stability, and surface chemistry [1,68]. In the present study, the potential cytotoxic (antimitotic) and genotoxic effects of AgNPs and bark extracts used for their synthesis were evaluated using *Allium cepa* assay. The latter is a simple, reliable, and widely used test for assessing the cytogenotoxicity of a product [69]. The effects of AgNPs and bark extracts on cell division and chromosome behaviour in *Allium cepa* cells are summarized in Figure 8 and Figure 9 and Table 3. *Picea abies* and *Pinus nigra* bark extracts and their derived AgNPs significantly decreased the number of dividing cells and consequently the mitotic index. The cytotoxic effects of AgNPs were more pronounced than those produced by the bark extracts. The mitotic index values of *Allium cepa* cells exposed to *Picea abies* and *Pinus nigra* bark extract derived AgNPs (1.27 ± 0.16% and 1.67 ± 0.09%, respectively) were almost half of those corresponding to the bark extracts (2.80 ± 0.17% and 2.97 ± 0.14%, respectively) and several-fold (6.18- and 4.7-fold, respectively) lower than the control (7.86 ± 0.23%). The percentages of cells in different mitotic phases were also calculated. The results clearly showed that AgNPs induced significant accumulation of cells in prophase (85.17 ± 0.77% and 84.32 ± 2.60% for *Picea abies* and *Pinus nigra* bark extract derived AgNPs, respectively) in comparison with the bark extracts (75.31 ± 2.32% and 67.40 ± 0.64% for *Picea abies* and *Pinus nigra* bark extracts, respectively) and control (53.80 ± 2.90%). AgNPs reduced the proportion of cells in metaphase, anaphase, and telophase in comparison with the control (Figure 8). The accumulation of cells in prophase induced by AgNPs and bark extracts can be ascribed to the inhibition of spindle fibers formation/function or G2 phase extension [22].

Plant derived AgNPs were reported to cause different types of chromosomal aberrations in *Allium cepa* cells such as chromosomal stickiness in metaphase and anaphase, unoriented or clumping chromosomes in disturbed metaphase, bridges, vagrant and lagging chromosomes in anaphase-telophase, chromosome fragmentation, in addition to micronucleus at interphase and C-mitosis [22,69,70,71]. In our study, *Picea abies* and *Pinus nigra* bark extract derived AgNPs were found to induce anaphase-telophase chromosomal aberrations such as vagrant chromosomes (in 1.05 ± 0.13% and 0.80 ± 0.25% of the total dividing cells, respectively) and multiple bridges (in 0.50 ± 0.06% and 0.55 ± 0.07% of the total dividing cells, respectively) (Table 3, Figure 9). Vagrants are chromosomes moving ahead of their associated chromosomal groups toward cell poles. Thus, the chromosomes in daughter cells are unequally separated resulting in polyploidy and aneuploidy. Chromosomal bridges usually result from the non-disjunction of sticky chromosomes or dysfunctions in separation (breakage followed by unification) during anaphase leading to change or loss of genetic material [72,73].

Overall, *Picea abies* and *Pinus nigra* bark extract derived AgNPs depressed mitosis arresting cell division at prophase stage and induced vagrant chromosomes and multiple bridges to a greater degree than the corresponding bark extracts. In previous studies, the cytogenotoxic potential of plant derived AgNPs was assessed using different versions of *Allium cepa* assay. AgNPs derived from *Melissa officinalis* (lemon balm) ethanolic extracts (10% and 20%) attenuated the mitodepressive effects of the extracts used for their synthesis as revealed by a several-fold enhancement in the mitotic index values but caused an increase in the number of vagrants (2.8- and 1.5-fold, respectively) in comparison with the corresponding extracts. AgNPs synthesized with 10% *M. officinalis* ethanolic extract increased the number of micronuclei (2.5-fold), too [22]. When comparing with the control, AgNPs derived from cocoa pod husk and cocoa bean extracts significantly reduced the mitotic index (1.8–10.7-fold) with prophase cell accumulation (2.84–10.55% increase in prophase cells) and induced C-mitosis, chromosomal bridges, sticky and vagrant chromosomes (0.06–0.48%) [71]. Cell laggards, disturbed metaphases with clumping chromosomes and disturbed telophases were reported for other plant derived AgNPs [69]. It can be concluded that *Picea abies* and *Pinus nigra* bark extract derived AgNPs showed antimitotic potential which could be exploited for the development of formulations with antitumor activity. Similar to other AgNPs derived from plant extracts, AgNPs obtained in the present study induced chromosomal aberrations but they occurred at low frequencies (≤ 1.05% of the total dividing cells).

## 3. Materials and Methods

### 3.1. Chemicals and Reagents

Folin-Ciocalteu’s phenol reagent, gallic acid, sodium carbonate, silver nitrate (AgNO_3_), carmine powder, hydrochloric acid 37%, 1-butanol, catechin, epicatechin, formic acid and acetonitrile (LC grade), water (LC grade) were purchased from Sigma-Aldrich (Steinheim, Germany). Ammonium iron (III) sulphate dodecahydrate was purchased from Riedel-de-Haën (Seelze, Germany). Absolute ethanol and glacial acetic acid were supplied by Chimreactiv (Bucharest, Romania). Mueller-Hinton agar, gentamicin (10 µg/disk) and nystatin (100 units/disk) came from Oxoid (Basingstoke, UK). Potato dextrose agar came from Bio-Rad (Hercules, CA, USA). Ultrapure water was obtained from SG Water Ultra Clear TWF water purification system (Barsbüttel, Germany).

### 3.2. Microorganisms

*Staphylococcus aureus* ATCC 25293, ATCC 33591 and ATCC 43300, *Staphylococcus epidermidis* ATCC 12228, *Streptococcus pyogenes* ATCC 19615, *Escherichia coli* ATCC 25922, *Pseudomonas aeruginosa* ATCC 9027 and *Candida albicans* ATCC 90028 were purchased from the American Type Culture Collection (ATCC, Manassas, VA, USA).

### 3.3. Plant Material and Extraction

*Picea abies* and *Pinus nigra* bark fragments were sampled from a natural site located in Suceava county, Northeast Romania (47°33′45″ N, 25°39′36″ E, elevation 734 m) in March 2018. For each species, five full-grown trees (35–40 years old, 18–25 cm circumference at breast height) were randomly selected for collection. The plant material was identified and authenticated by Dr. Constantin Nechita (Forest Research and Management Institute, Campulung Moldovenesc, Suceava, Romania). Voucher samples (PA1503/2018, PN1603/2018) are deposited in the Department of Pharmacognosy, Faculty of Pharmacy, Grigore T. Popa University of Medicine and Pharmacy, Iasi, Romania.

Bark fragments were thoroughly rinsed with distilled and ultrapure water, chopped, dried at room temperature, under ventilation and low luminosity and ground to obtain a homogenous fine powder. Bark powders were extracted as previously described with minor changes [40]. In brief, 10 g of bark powder of *Picea abies* and *Pinus nigra* were mixed with 100 mL of ultrapure water at 60 °C and stirred by magnetic stirrer (400 rpm) for 3 h at room temperature. The extracts were filtered (Whatman no. 1 filter paper), adjusted to a volume of 100 mL with ultrapure water and stored at −20 °C until further use.

### 3.4. Total Phenolic Content

The total phenolic content of *Picea abies* and *Pinus nigra* bark extracts was determined using the Folin-Ciocalteu method as previously described [67]. The total phenolic content was expressed as mg gallic acid/mL extract. The assay was performed in triplicate and the results were expressed as mean ± standard deviation.

### 3.5. Total Proanthocyanidin Content

The total proanthocyanidin content was estimated by the butanol-hydrochloric acid assay as previously reported and expressed as cyanidin equivalents (mg cyanidin/mL extract) using the molar extinction coefficient of cyanidin (ε = 17,360 L·mol^−1^·cm^−1^) [67,74]. The assay was done in triplicate and the results were expressed as mean ± standard deviation.

### 3.6. HPLC-DAD-ESI-Q-TOF-MS/MS Analysis

The phenolic profile in both bark extracts was analysed by HPLC-DAD-ESI-Q-TOF-MS/MS. The analysis was performed on an Agilent 1200 HPLC system (Agilent Technologies, Santa Clara, CA, USA) equipped with auto-sampler (G1329B), degasser (G1379B), binary pump (G1312C), thermostat (G1316A), DAD detector (G1315D), Agilent ESI-Q-TOF mass spectrometer (G6530B), nitrogen generator (Parker Hannifin Corp., Cleveland, OA, USA) and compressed air generator (Jun-Air Oxymed, Łódź, Poland). A Phenomenex Gemini C18 (100 × 2 mm, 3 µm) column was used. The mobile phase consisted of 0.1% formic acid in water (A) and 0.1% formic acid in acetonitrile (B). The elution gradient, injection volume, flow rate, column temperature and ESI-Q-TOF-MS parameters were those reported by Luca et al. [31], except that the mass range varied from 100 to 1700 *m*/*z*. Data were processed with a MassHunter Qualitative Analysis Navigator B.08.00 software (Agilent).

### 3.7. Green Synthesis of Colloidal Silver Nanoparticles

For the green synthesis of AgNPs, *Picea abies* and *Pinus nigra* aqueous bark extracts (1 mL of each) were added to 100 mL of 1 mM AgNO_3_. After stirring for 60 min at room temperature, a stable reddish-brown colour appeared indicating the formation of AgNPs [40].

### 3.8. UV-Vis Spectroscopy

The formation of AgNPs was monitored by recording the UV-Vis spectrum of each reaction mixture in the wavelength range of 300–700 nm [47,58] on a Specord 210 Plus spectrophotometer (Analytik Jena, Jena, Thuringia, Germany). Samples were collected at different times (0, 5, 10, 15, 20, 25, 30 and 60 min) after the start of stirring but also at 2, 3, 4, 24 and 48 h after the end of stirring. At the aforementioned times, 1 mL of the reaction mixture was sampled and further diluted with 2 mL of ultrapure water in quartz cuvettes followed by recording the UV-Vis spectrum.

### 3.9. Determination of Concentration of Silver Nanoparticles

The concentration of AgNPs in colloidal solution was determined according to previous reports. In brief, the average number of atoms per nanoparticle (*N*) was first calculated as follows:

(1) $$N=\frac{\pi \rho D^{3}N_{A}}{6M}\;$$

where *π* = 3.14, *ρ* = 10.5 g/cm^3^ (density of face centered, cubic Ag), *D* = average diameter of nanoparticles (in cm), *M* = 107.868 g (atomic mass of Ag) and *N_A_* = 6.023 × 10^23^ mol^−1^ (Avogadro’s number). Assuming that Ag^+^ completely converted to AgNPs, the molar concentration of the nanoparticle solution (C) was determined:

(2) $$C=\frac{N_{T}}{NVN_{A}}$$

where *N_T_* = total amount of Ag atoms added as AgNO_3_ (1.0 mM = 0.001 M), *N* = average number of atoms per nanoparticle, *V* = reaction volume (0.101 L) and *N_A_* = 6.023 × 10^23^ mol^−1^ (Avogadro’s number) [75,76].

### 3.10. ATR-FTIR Spectroscopy

ATR-FTIR spectra of AgNPs and bark extracts were obtained on a Bruker Alpha-P ATR FTIR spectrometer with diamond crystal (Bruker, Ettlingen, Germany). Prior to analysis, the colloidal AgNPs solutions were centrifuged at 7000 rpm for 20 min (Rotina 380 R centrifuge, Hettich, Tuttlingen, Germany). The pellets were resuspended in ultrapure water and subjected again to centrifugation (7000 rpm, 20 min) to eliminate unreacted AgNO_3_ and other free compounds that were not involved in the stabilization of AgNPs. The pellets were further freeze dried (Unicryo TFD 5505 freeze dryer, UniEquip GmbH, Munich, Germany). Bark extracts (30 mL of each) were concentrated under reduced pressure at 40 °C (Büchi R-210 rotary evaporator system, Büchi Labortechnik AG, Flawil, Switzerland) and freeze-dried. ATR-FTIR measurements were performed with a resolution of 4 cm^−1^ in the spectral region 4000–400 cm^−1^. Data were processed using Bruker OPUS spectroscopy software ver. 7 ed. 2011.

### 3.11. Raman Spectroscopy

Raman spectra of colloidal AgNPs solutions were recorded using an inVia confocal Raman microscope (Renishaw, Wotton-under-Edge UK) equipped with a high-power near-infrared diode laser at 785 nm (300 mW) and a Charge Coupled Device (CCD) detector coupled to a Leica DM2500 M microscope. All measurements were performed in the backscattering geometry using a 50× objective (numerical aperture 0.75, working distance 0.37 mm), at room temperature and atmospheric pressure. Scans were accumulated to obtain spectra in the wavelength domain 100–3500 cm^−1^ with a resolution of 1 cm^−1^. Spectral manipulations were performed with WiRE 3.2 software (Renishaw, Wotton-under-Edge, UK).

### 3.12. DLS Analysis

DLS analysis was performed to determine the hydrodynamic diameter (Z-average), PDI and zeta potential values of AgNPs (Malvern Zetasizer Nano-ZS, Malvern Instruments, Malvern, UK). For the hydrodynamic diameter and PDI measurements, the samples were analysed at 25 °C by DLS at an angle of 90°, using red He/Ne laser at λ = 633 nm. Zeta potential was recorded through electrophoretic light scattering at 25 °C.

### 3.13. SEM and EDX Analysis

The experiments were performed with a Quanta 200 scanning electron microscope equipped with an energy dispersive spectrometer (FEI Company, Hillsboro, OR, USA). The samples were fixed on adequate support and operated in low vacuum mode at 20 kV. The AgNPs surface was observed under different magnifications with a secondary electron detector (large field detector) while a silicon drift detector was used to obtain elemental information.

### 3.14. TEM Analysis

The samples were examined in the high contrast mode on a Hitachi High-Tech HT7700 transmission electron microscope (Hitachi High-Technologies Corporation, Tokyo, Japan) at 120 kV accelerating voltage. The instrument is equipped with a Bruker EDX detector that allows elemental analysis and a SAED aperture that could be used to collect diffraction patterns directly during the sample inspection. A drop of each colloidal AgNPs solution was placed on a 300-mesh carbon-coated copper grid (Ted Pella) and vacuum-dried at room temperature for 24 h prior to examination.

### 3.15. Antimicrobial Assay

The antimicrobial activity of AgNPs and bark extracts used for their synthesis was investigated by the disk diffusion method against Gram-positive and Gram-negative bacteria (*S. aureus*, *S. epidermidis*, *S. pyogenes* and *E. coli*, *P. aeruginosa*, respectively) and fungi (*C. albicans*). In brief, microbial inoculum, adjusted to the turbidity of 0.5 McFarland standard, was spread over Mueller-Hinton agar (for bacteria) and potato dextrose agar (for fungus) in sterile Petri plates (diameter 9 cm). Sterile paper disks (diameter 6 mm), impregnated with equal volumes of colloidal AgNPs solutions and bark extracts (25 µL) were placed over medium. The diameters of the inhibition zones were measured after 24 h incubation at 37 °C [4,77]. Gentamicin (10 µg/disk) and nystatin (100 units/disk) were used as positive controls. Each experiment was performed in triplicate and the results were expressed as mean ± standard deviation.

### 3.16. Cytogenotoxicity Assay

The cytogenotoxicity of AgNPs and bark extracts was assessed by *Allium cepa* assay as previously described [22,71,78] with some modifications. High quality equal-sized onion bulbs were purchased from a local market. Their outer scales were removed, and their bottoms were scraped to expose root primordia. Onion bulbs were further placed in 100 mL jars filled with tap water, only the root primordia being immersed into water. After 24 h, the tap water in the jars was replaced with the same volume of each sample. The root primordia were kept immersed in samples for another 12 h. The control was represented by the onion bulbs immersed in tap water for 36 h. For each sample and control, the root tips of three onions were sampled, the roots from each bulb being processed separately. The roots were initially fixed in ethanol:glacial acetic acid (3:1, *v*/*v*) for 12 h at 4 °C and then heated for 20 min in acetic carmine solution (10 mL) containing ten drops of 1 N hydrochloric acid. The root tips were cut on a glass slide, covered with a coverslip, and squashed. A number of approximatively 3000 cells per each slide were examined for the mitotic index, mitotic stages and chromosome aberrations using an Optika B-159 microscope (400× magnification) coupled with Optikam B5 camera (Image J program) (Optika, Ponteranica Italy). The mitotic index was calculated as the percentage of cells undergoing mitosis of the total number of cells scored. The number of cells in different mitotic stages (prophase, metaphase, anaphase, telophase) was expressed as percentage of the total dividing cells. In the same manner (percentage of the total dividing cells), different types of chromosomal aberrations were also quantified. All experiments were performed in triplicate and the results were expressed as mean ± standard deviation.

### 3.17. Statistical Analysis

Statistical analyses were performed using SPSS software version 18.0. The paired samples *t*-test was used for comparing the means of any two normally distributed variables.

## 4. Conclusions

In the present study, colloidal phyto-functionalized AgNPs were produced using bark aqueous extracts of *Picea abies* and *Pinus nigra*. For both bark extracts, polyphenols were found to be majorly involved in the synthesis and stabilization of AgNPs but ATR-FTIR and Raman spectroscopic data clearly indicated the contribution of other compounds in bark extracts (carbohydrates, proteins). DLS, SEM, TEM and EDX techniques showed that *Picea abies* and *Pinus nigra* bark extract derived AgNPs were spherical in shape, crystalline, homogenous, and well dispersed, with mean hydrodynamic diameters below 100 nm which is a prerequisite for a good antimicrobial activity. Both AgNPs exhibited stronger antibacterial, antifungal, and antimitotic effects than the bark extracts used for their synthesis. Based on these results, future investigations will focus on the development of novel formulations by impregnation of the colloidal AgNPs solutions obtained in the present study into polymeric nanofibers for topical application in infectious and hyperproliferative skin disorders. In addition, both *Picea abies* and *Pinus nigra* are used in the lumber industry, the bark being the main waste. The green synthesis of AgNPs could be a potential route for the valorization of bark waste.

## Figures and Tables

**Table 1:** Compounds tentatively identified in *Picea abies* and *Pinus nigra* aqueous bark extracts.

No.	T_R_ [min]	[M − H]^−^ [*m*/*z*]	MF	MS/MS Fragments [*m*/*z*]	Proposed Identity	Sample	Ref.
1	1.9	191.0561	C_7_H_12_O_6_	173.0437; 127.0442	Quinic acid	*Picea abies* *Pinus nigra*	[24]
2	2.5	191.0204	C_6_H_8_O_7_	129.0237; 111.0126	Citric acid	*Picea abies* *Pinus nigra*	[24]
3	7.0	329.0830	C_14_H_18_O_9_	167.0425; 152.0186; 123.0506	Vanillic acid hexoside	*Pinus nigra*	[25]
4	8.1	345.1533	C_16_H_26_O_8_	183.0293; 139.0322	Methoxy- dihydroxybenzoic acid hexoside	*Picea abies*	[26]
5	8.5	299.0832	C_13_H_16_O_8_	137.0284	Hydroxybenzoic acid hexoside	*Pinus nigra* *Picea abies*	[24]
6	8.9	315.0995	C_14_H_20_O_8_	153.0553; 109.0301	Dimethoxyphenyl hexoside	*Picea abies* *Pinus nigra*	[27]
7	9.4	313.0919	C_14_H_18_O_8_	151.0433	Vanillin hexoside	*Pinus nigra*	[28]
8	9.8	137.0257	C_7_H_6_O_3_	109.0245	Hydroxybenzoic acid	*Picea abies* *Pinus nigra*	[24,25]
9	12.5	343.1385	C_16_H_24_O_8_	181.1133	Dihydro- coniferylalcohol hexoside I	*Pinus nigra*	[29,30]
10	14.0	337.0939	C_16_H_18_O_8_	191.0561; 163.0430; 119.0516	*p*-Coumaroylquinic acid	*Pinus nigra*	[31]
11	14.4	577.1331	C_30_H_26_O_12_	451.1057; 425.0931; 407.0825; 289.0747; 245.0451; 125.0268	Procyanidin dimer	*Picea abies* *Pinus nigra*	[32,33]
12	14.6	465.1025	C_21_H_22_O_12_	303.0651; 285.0537; 259.0775; 125.0363	Taxifolin hexoside I	*Pinus nigra*	[32,34]
13	14.8	355.1050	C_16_H_20_O_9_	193.0604; 178.0266; 149.0626	Ferulic acid hexoside	*Picea abies*	[35,36]
14	15.3	327.1058	C_15_H_20_O_8_	165.0553	Dihydroxy propiophenone hexoside	*Picea abies*	[37]
15	15.8	289.0778	C_15_H_14_O_6_	271.0678; 245.0727; 205.0661; 151.0330	Catechin *	*Picea abies* *Pinus nigra*	[25,32]
16	16.3	343.1388	C_16_H_24_O_8_	181.1149	Dihydro coniferylalcohol hexoside II	*Pinus nigra*	[29,30]
17	17.3	865.1959	C_45_H_38_O_18_	577.1237; 407.0348; 289.0753	Procyanidin trimer	*Pinus nigra*	[24,32]
18	19.4	289.0765	C_15_H_14_O_6_	271.0666; 245.0733; 205.0654; 151.0311	Epicatechin *	*Pinus nigra*	[24,25]
19	20.5	405.1172	C_20_H_22_O_9_	243.0723; 201.0586; 159.0482	Piceatannol hexoside I	*Picea abies*	[38]
20	21.9	465.1025	C_21_H_22_O_12_	303.0638; 285.0539; 259.0749; 125.0313	Taxifolin hexoside II	*Picea abies * *Pinus nigra*	[32,34]
21	22.5	405.1169	C_20_H_22_O_9_	243.0733; 201.0533; 159.0466	Piceatannol hexoside II	*Picea abies*	[38]
22	23.2	389.1236	C_20_H_22_O_8_	227.0745; 185.0652; 143.0537	Resveratrol hexoside	*Picea abies*	[38]
23	23.5	303.0575	C_15_H_12_O_7_	285.0469; 259.0696; 125.0279	Taxifolin	*Picea abies* *Pinus nigra*	[32,34]
24	23.8	419.1329	C_21_H_24_O_9_	257.0856	Isorhapontigenin hexoside	*Picea abies*	[38]
25	25.1	243.0619	C_14_H_12_O_4_	215.0696; 201.0594; 109.0280	Piceatannol	*Picea abies*	[38]
26	26.1	447.1009	C_21_H_20_O_11_	301.0323; 255.0569	Quercetin rhamnoside	*Picea abies*	[25,33]
27	26.4	809.2260	C_40_H_42_O_18_	647.1892; 405.1233; 243.715	Piceaside A/B/G/H	*Picea abies*	[38]
28	26.9	837.2621	C_42_H_46_O_18_	675.2224; 513.1569; 243.0739	Piceaside O/P	*Picea abies*	[39]
29	28.9	823.2461	C_41_H_44_O_18_	661.2020; 499.1511; 403.0937; 241.0567	Piceaside E/F	*Picea abies*	[38]
30	29.8	647.1749	C_34_H_32_O_13_	585.2230; 485.1132; 451.1132	Piceaside J/K	*Picea abies*	[38]
31	30.2	257.0827	C_15_H_14_O_4_	241.0521; 224.0468	Isorhapontigenin	*Picea abies*	[38]
32	30.7	647.1778	C_34_H_32_O_13_	485.1267; 405.1142; 243.0645	Piceaside I/J/K	*Picea abies*	[38]

MF: molecular formula; T_R_: retention time; * confirmed with standard.

**Table 2:** Diameters of inhibition zones (mm) developed by AgNPs and bark extracts used for their synthesis against pathogenic bacteria and fungi.

Microorganism	Nystatin	Gentamicin	*P. abies*- AgNPs	*P. abies* Extract	*P. nigra*- AgNPs	*P. nigra* Extract
*S. aureus* ATCC 25293	ND	23.00 ± 0.58 ^nc^	14.67 ± 0.58 ^nc,a^	13.00 ± 1.73 ^nc,a,b^	16.00 ± 2.64 ^nc,a,b,a^	NA
*S. aureus* ATCC 33591 (MRSA)	ND	15.67 ± 0.58 ^nc^	15.67 ± 1.15 ^nc,c^	10.67 ± 0.58 ^nc,a,a^	14.67 ± 0.58 ^nc,b,b,a^	10.67 ± 1.15 ^nc,a,a,c,b^
*S. aureus* ATCC 43300 (MRSA)	ND	7.67 ± 0.58 ^nc^	15.33 ± 0.58 ^nc,a^	7.67 ± 0.58 ^nc,c,a^	15 ± 1.0 ^nc,a,c,a^	NA
*S. epidermidis* ATCC 12228	ND	29.67 ± 0.58 ^nc^	19.00 ± 1.73 ^nc,a^	14.67 ± 1.15 ^nc,a,a^	16 ± 1.0 ^nc,a,a,b^	12 ± 0.0 ^nc,a,a,b,a^
*S. pyogenes* ATCC 19615	ND	18.33 ± 1.15 ^nc^	12.67 ± 0.58 ^nc,b^	NA	13 ± 1.0 ^nc,a,c,nc^	NA
*E. coli* ATCC 25922	ND	20.67 ± 0.58 ^nc^	14 ± 1 ^nc,a^	NA	16.33 ± 2.08 ^nc,a,b,nc^	NA
*P. aeruginosa* ATCC 9027	ND	23.33 ± 0.58 ^nc^	13 ± 1 ^nc,a^	NA	13.67 ± 0.58 ^nc,a,c,nc^	NA
*C. albicans* ATCC 90028	21.33 ± 0.58	ND ^nc^	16.25 ± 1.73 ^a,nc^	7.91 ± 1.15 ^a,nc,a^	14.67 ± 1.52 ^a,nc,b,a^	9.33 ± 1.15 ^a,nc,a,b,a^

ND: not determined; NA: no activity; ^nc^: not computed; ^a^: *p* < 0.001; ^b^: *p* < 0.05; ^c^: no significance.

**Table 3:** Impact of *Picea abies* and *Pinus nigra* bark extracts and their derived AgNPs on chromosomal aberrations in *Allium cepa* root meristems.

Sample	Vagrants (%)	Multiple Bridges (%)	Interrupted Bridges (%)
Control	0.06 ± 0.04	-	-
*Picea abies* bark extract	0.31 ± 0.13 ^a^	-	0.27 ± 0.15
*Pinus nigra* bark extract	0.45 ± 0.22 ^a,a^	0.26 ± 0.08	-
*Picea abies* bark extract derived AgNPs	1.05 ± 0.13 ^a,a,a^	0.50 ± 0.06 ^a^	-
*Pinus nigra* bark extract derived AgNPs	0.80 ± 0.25 ^a,a,a,a^	0.55 ± 0.07 ^a,a^	-

^a^: *p* < 0.001.

## Data Availability

Data supporting reported results are available from the corresponding authors.

## Sample Availability

Samples of the compounds are available from the authors.

## Abbreviation

AgNPs	Silver nanoparticles
ATR-FTIR	Attenuated total reflection Fourier-transform infrared
DLS	Dynamic light scattering
EDX	Energy dispersive X-ray analysis
HPLC-DAD-ESI-Q-TOF-MS/MS	High-performance liquid chromatography with diode array detection coupled to electrospray ionization quadrupole time-of-flight tandem mass spectrometry
MF	Molecular formula
PDI	Polydispersity index
MRSA	Methicillin-resistant *Staphylococcus aureus*
MSSA	Methicillin-susceptible *Staphylococcus aureus*
SAED	Selected area electron diffraction
SEM	Scanning electron microscopy
TEM	Transmission electron microscopy
T_R_	Retention time
Z-average	Hydrodynamic diameter
